# Results of TcMEP guided vestibular schwannoma surgery; long term follow-up and progression free survival

**DOI:** 10.1007/s10143-025-04072-1

**Published:** 2026-02-07

**Authors:** Zora A. Gorissen, Juerd Wijntjes, Henricus P. M. Kunst, Mark ter Laan

**Affiliations:** 1 Department of Neurosurgery, Radboud University Medical Center, Nijmegen, The Netherlands; 2 Department of Neurology, Radboud University Medical Center, Nijmegen, The Netherlands; 3 Department of Otorhinolaryngology, Radboud University Medical Center, Nijmegen, The Netherlands; 4 Department of Otorhinolaryngology, Maastricht University Medical Centre, Maastricht, the Netherlands; 5 Dutch Academic Alliance Skull Base Pathology, Radboud University Medical Centre, Maastricht University Medical Centre+, Nijmegen, Maastricht, the Netherlands

**Keywords:** Facial nerve, Vestibular schwannoma, Intraoperative neuromonitoring, Recurrence

## Abstract

In patients with large vestibular schwannomas (VS), surgery is the primary treatment despite risks such as facial nerve dysfunction. Intra-operative monitoring with transcranial motor evoked potentials (TcMEP) helps predict facial nerve outcome but may lead to subtotal resections and later recurrence. This study compares TcMEP thresholds, facial nerve outcome, recurrence rate and progression-free survival in Koos grade 4 vestibular schwannomas. Fifty-five surgically treated patients with Koos grade 4 VS (2015–2024) were included in this retrospective study. All underwent facial nerve TcMEP monitoring. House-Brackmann (HB) scores were assessed postoperatively, and at 6 weeks, 6 months and 1 year. Progression-free survival was analyzed with Kaplan–Meier curves. Postoperatively, 58% showed a decline in facial nerve function. After one year, 83% had good function (HB I–II). A TcMEP threshold increase < 20 mA correlated with good outcomes. In 84% (47/55), a small residual tumor remained (mean 0.4 cc; range 0–7.9 cc). Median growth-free survival was 76 months after subtotal resection (STR) and not reached after near-total resection (NTR). We conclude that TcMEP-guided surgery for large vestibular schwannomas provides good facial nerve outcomes, small acceptable remnants and a low long-term recurrence risk. As fewer than half of residual tumors show growth within 8 years, routine postoperative radiotherapy is not recommended; a watchful-waiting strategy is preferred.

## Introduction

Five to ten percent of all intracranial tumors are located in the cerebellopontine angle (CPA) region [1]. The most common CPA neoplasms are vestibular schwannomas (VS), derived from myelinating Schwann cells of the vestibulocochlear (eighth cranial) nerve, and meningiomas [2]. Most often - in case of smaller tumors (< 3 cm diameter) and no oedema/hydrocephalus - a regimen of ‘wait and scan’ or single-fractionated stereotactic radiosurgery (SRS) is justified. In larger tumors, (diameter over 3 cm, Koos classification grade 4, indicating brainstem/cerebellar compression) surgery is the first choice of treatment despite the risk of complications such as loss of facial nerve (FN) function [2, 3].

To reduce the risk for this complication, intra-operative neuromonitoring (IOM) of the facial nerve is applied during surgery [4]. One of the most used IOM techniques is measuring transcranial motor evoked potentials (TcMEP), either with the supramaximal or the threshold method, even though standardization is still out for debate [5]. Previous research has shown that TcMEP, especially with the threshold method, is a valuable predictor for FN outcome [4, 6, 7]. A TcMEP threshold change (deltaMEP) of less than 20 mA was correlated with preservation of good FN function (House-Brackmann grade (HB) I or II after 6 months in 73% of patients) [6, 7]. Therefore, using this threshold change may serve as a guide for safe resection of CPA tumors and preserve facial nerve function.

However, a drawback may be that this leads to subtotal resections, indicating more residual tumor, and an increased risk of recurrence over time. Although historically, gross total resection (GTR) of VS was the first choice as a surgical strategy, current literature demonstrates that subtotal resection may benefit patients quality of life by preservation of the FN, although the definition of near and subtotal varies along reports [8, 9].

This constitutes an ongoing debate, where one must balance between extent of resection (EOR), preservation of FN function and progression free survival (PFS) over time. Besides reaffirming the correlation between TcMEP threshold change and post-operative FN function, this study is aimed at finding the relation between residual tumor and recurrence rates in vestibular schwannoma patients that underwent surgery. Two questions will be addressed: (1) does TcMEP threshold change predict FN outcome in this cohort with extended follow-up? And (2) what are the recurrence rates of VS when using TcMEP guided surgery?

## Methods

Data was recorded on 90 patients with a CPA tumor that required (first time) surgery at the Radboud University Medical Center (RUMC) between February 2015 and March 2024 in Nijmegen, The Netherlands. In alignment with Dutch law no ethical approval is required for retrospective studies. Research is performed in accordance with the Declaration of Helsinki. Informed consent for use of their data was obtained of all individual participants.

Data regarding demographics, laterality, clinical presentation, tumoral characteristics, intra-operative findings, postoperative FN function, complications, tumoral growth and follow-up were recorded. IOM data was recorded perioperatively. Final tissue diagnosis was done by the pathologist. Typical follow-up consisted of a consultation around 6 weeks post-operatively, around 6 months, and then yearly to bi-annually. Postoperative MRI was performed at 3 months, 15 months, and subsequently bi-annually or according to surgeon’s judgement.

In all procedures a standard retrosigmoid approach was used with the patient in a supine position, the head turned and tilted towards the contralateral side. Surgery was under total intravenous anaesthesia, guided by multimodal IOM (including electromyography and TcMEP) as described in our previous study [6]. Needle electrodes were placed in the ipsilateral orbicularis oculi, orbicularis oris, nasalis and mentalis muscles. A single pulse was used to check for direct (peripheral) stimulation of the facial muscles, followed by a train of five to elicit a corticobulbar facial MEP response. The threshold-level method for TcMEP was used resulting in reliable measurements as described previously by Hendriks et al. and Sarnthein et al. (2013). A 20 mA threshold increase has been shown to provide highest predictive value for postoperative facial nerve dysfunction. Sudden intensity changes are assumed to reflect pathological alterations of the facial nerve [6, 7].

TcMEP stimulation baselines were set before incision and checked after dural opening. Baseline TcMEP threshold was determined in steps of 5 mA until a MEP was detected in one of the target muscles of the facial nerve. A MEP response as low as 50 µV with appropriate response latency was qualified as a reliable MEP response. The testing was repeated every 30s. Movement artifacts are minimal with this technique and were always discussed with the surgeon. The surgical procedure was never interrupted for TcMEP stimulation due to the minimal extent of movement. The first dorsal interosseous muscle was monitored as a control to account for systemic influences on TcMEP amplitude, such as anesthesia and blood pressure. As of note, without supramaximal stimulation, percentage decreases and latency changes could not be assessed. Additionally, the trigeminal nerve and accessory nerves were monitored, and glossofaryngeus and vagus nerves when applicable. When hearing was (partly) intact, brainstem auditory evoked potential (BAEP) was measured during surgery, although almost all patients were functionally deaf.

Throughout the surgical procedure, direct nerve stimulation (DNS) was frequently employed to identify and confirm the facial nerve’s trajectory, with stimulation intensity ranging from 0.05 to 0.5 mA with an anodal pulse of 200 µs. Sudden intensity increases, reaching the 20 mA intensity threshold and sudden extensive A-trains during a surgical event were directly communicated to the surgeon. The decision to continue or terminate surgery (resulting in subtotal or near total resection) depended on factors such as TcMEP recovery, spontaneous EMG responses (including A-trains) and the anatomical view of the facial nerve. If the TcMEP threshold reached 20 mA or more and did not improve, the surgery was terminated, unless the last bit of tumor could be taken out with minimal manipulation of the facial nerve. Complications were defined as new deficits within 30days post-surgery.

Tumor growth was defined as a significant increase in rest size determined by the radiologist (> 2 mm in one axial direction). Tumor volumes were measured using either T2 space or T1 post-contrast images. Segmentation was done semiautomatically Brainlab^®^ Elements software (Brainlab AG, Munich).

Rest size was defined as Gross Total Resection (GTR), with no detectable tumor rest size; Near Total Resection (NTR), with a tumor rest size of > 0 to 1 cm3; Sub Total Resection (STR) with a tumor rest size of > 1 cm3.

Statistical analyses was performed using IBM SPSS Statistics version 29. Correlations between HB grade score and TcMEP threshold change (DeltaMEP) were determined by Spearman’s correlation coefficient, and included all patients. PFS rates were analyzed using Kaplan-Meier (KM) survival curves. In this analysis, only non-NF (neurofibromatosis) VS patients were included in order to have a homogenous sample. A *p*-value ≤ 0.05 was considered statistically significant (95% confidence interval).

## Results

### Patient and tumor characteristics

A total of 90 patients with a CPA tumor between 2015 and March 2024 underwent first time surgery for a CPA lesion using IOM including TcMEP. Thirty-five were excluded: 7 patients did not give consent to use their data, 25 patients had other diagnoses then VS (15 meningioma, 4 jugular schwannoma, 3 epidermoid, 1 ependymoma, 1 lymphoma and 1 papilloma), 3 patients had NF related schwannomatosis.

Pre-operatively, eight patients showed some facial weakness; one presented with HB grade IV, two patients with grade 3 and seven patients with HB grade V. Forty-five patients had severe unilateral hearing loss.

The average age of patients at the time of surgery was 52 years (range: 17–82). About half was male and half was right sided, median tumour size and volume were 36 mm (largest axial parapetrosal diameter) and 14,1cm^3^ respectively (Table 1).

**Table 1:** Patient and tumour characteristics of the (non-NF) vestibular Schwannoma cases (*N* = 59)

Patient and tumour characteristics	*n* = 55
Average age	52 (17–82)
Male	28 (51%)
Right sided	27 (49%)
Median tumour size in mm	36 (19–53)
Median tumour size in cm^3^	14,1 (3,7–34,4)

**Table 2:** Surgery results for Non-NF II VS patients. GTR = no remnant, NTR = 0-1 cc remnant, STR = ≥ 1 cc remnant

Surgical results	Non-NF VS patients (*n* = 55) (min/max)
Average surgery time (minutes)	267 (134–476)
Median rest size (cm^3^)*	0,40 (0,0–7,91)
Median resection (%)*	97,2 (31,6-100)
Proportion GTR	8 (15%)
0 < Proportion NTR, < 1cc	30 (54%)
Proportion STR, = ≥ 1cc	17 (31)%

### Surgical results

The average operation time, was 4.5 h (range: 134–476 min) (Table 2).

GTR was achieved in 8/55 (15%) of patients, NTR was achieved in 30/55 (54%) of patients and STR in 16/55 (31%) of patients. Median tumor rest size was 0.4 cm^3^ (range: 0.0–7.91) with a median resection percentage of 97%.

### Complications

Post-operative complications are shown in Table 3. 58% showed decreased facial nerve functioning immediately post-operative (32 patients), 7 had loss of hearing or deteriorated hearing, others were deaf already pre-operatively.

**Table 3:** Complications in non-NF VS patients. VS = vestibular schwannoma, CSF = cerebral spinal fluid. * direct post-operative facial nerve function

Complications	Non-NF VS (*n* = 55)
None	15 (27%)
Facial nerve deficit (increase)*	32 (58%)
Trigeminal issues	8 (14%)
Minor dysphagia or hoarseness	4 (7%)
Hearing loss (increase)	7 (13%)
Balance/cerebellar deficit	5 (9%)
CSF leakage	4 (7%)
Diplopia	1 (2%)
Hydrocephalus	1 (2%)
Post-op Bleeding	0 (0%)
Other	1 (2%)

### IOM and facial nerve outcome

**Fig. 1:**
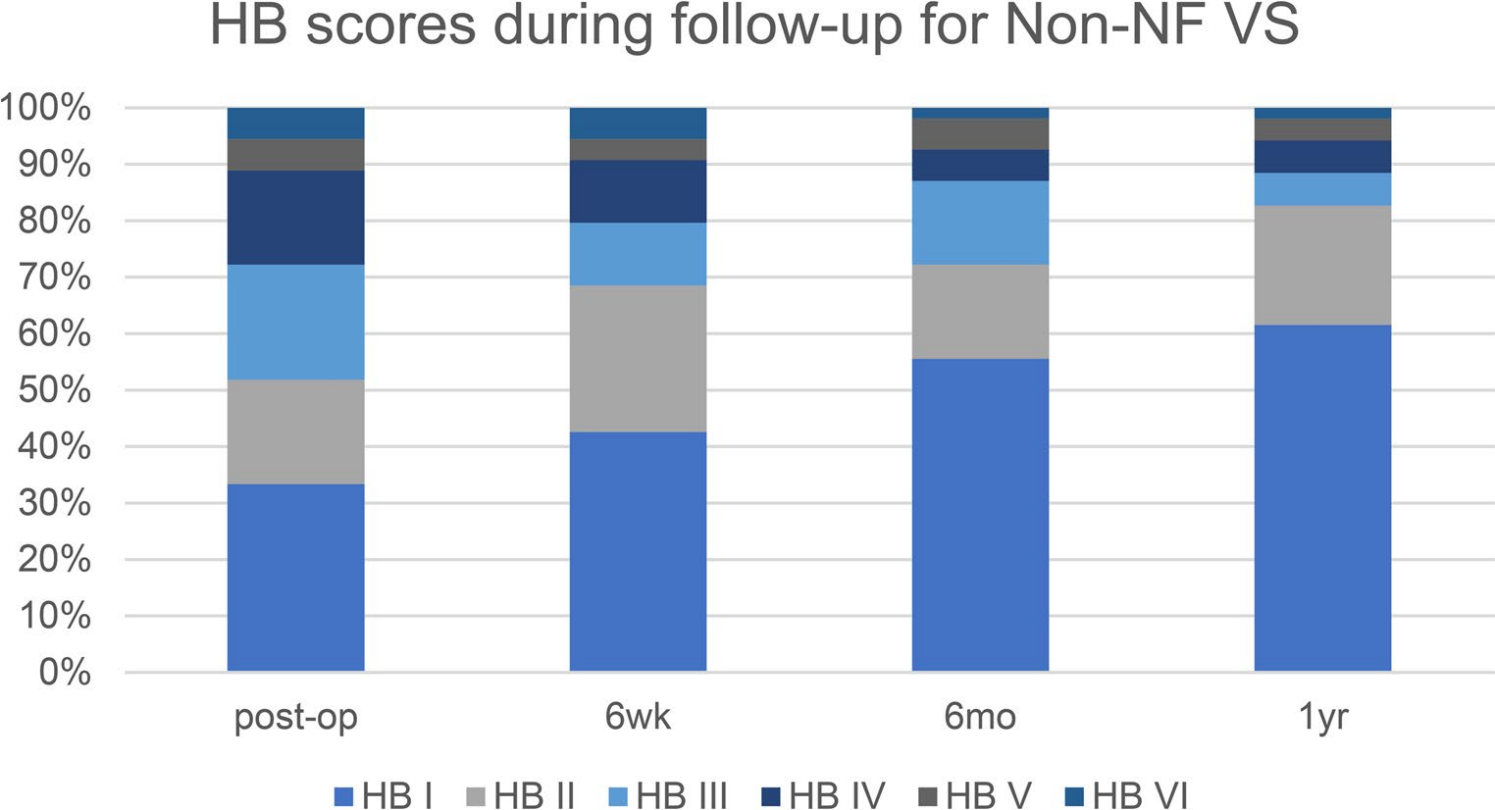
HB scores immediate post-op and at 6 weeks, 6 months post-op (*n* = 54) and 1 year post-op (*n* = 52)

Of all 55 Non-NF VS patients, in one no MEP could be achieved. In all but two 1 year follow-up data could be analyzed. Post-operative facial nerve function improved during follow-up as shown in Fig. 1. Good facial nerve outcome (HB I or II) was reached in 83% of patients and poor outcome (HB V or VI) in 5%.

### Threshold analysis

**Fig. 2:**
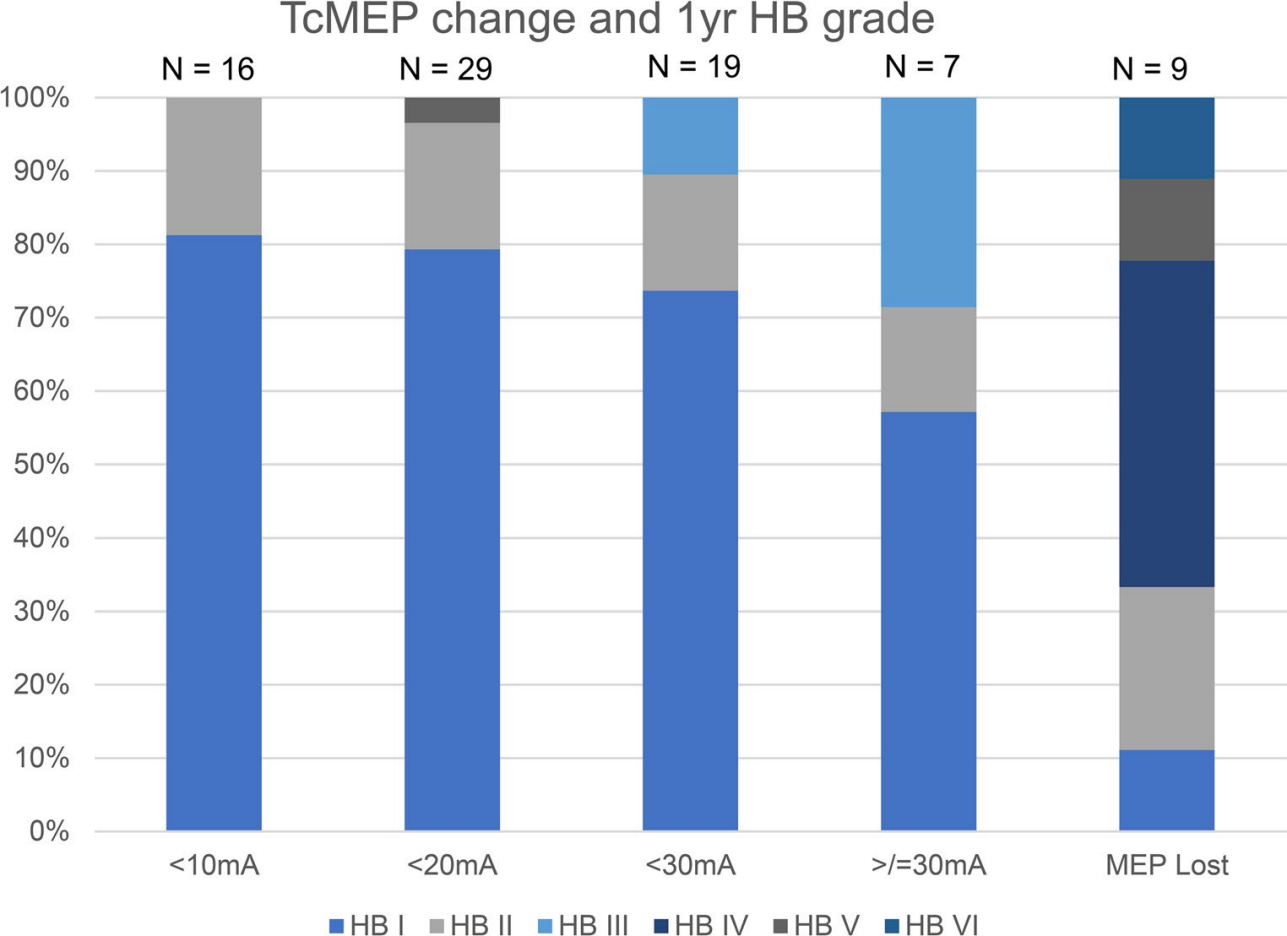
HB score at 1 year according to TcMEP threshold change. The only patient with HB > II with a thershold change of < 20 mA had a facial paresis of HB IV pre-op already. Results of all CPA tumour patients (including VS)

When looking at the distribution of HB grades over threshold changes, there are too few patients in each subgroup to analyze this for VS patients separately. When considering all CPA tumour patients treated in the same period (*n* = 90 of whom 80 could be analyzed), we can find that a threshold change of less than 20 mA never leads to a HB grade higher than II when facial nerve function is normal pre-op. The only case we found a worse outcome (HB V) already presented with a grade IV paresis (Fig. 2). Five patients showed an improved FN function (3 of them had VS).

This corroborates our previous findings concerning the use of the 20 mA threshold, even though correlation between the change in MEP threshold (deltaMEP) during surgery and FN function at one year was not as strong as previously reported (Spearmans correlation coefficient of 0.4) [6].

### Survival analyses

**Fig. 3:**
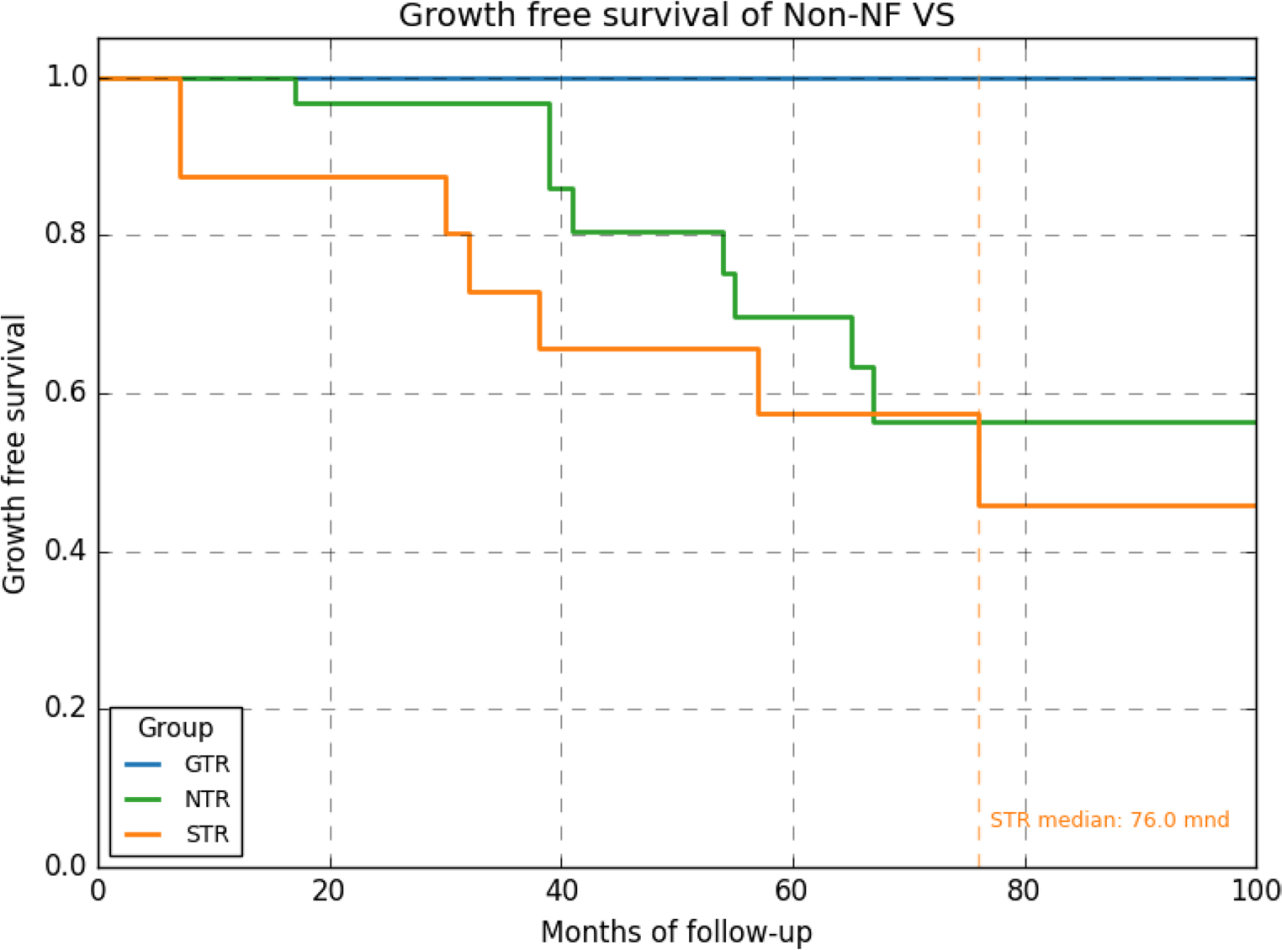
growth of recurrence over time in months based on extent of resection; GTR versus NTR and STR. No statistical significant difference was found between STR and NTR. Median growth free survival was 76 months for STR, not reached for NTR

Median follow-up was 83 months (time since surgery). None of the patients with GTR (*n* = 8) developed recurrence, 16 of the patients with a remnant developed recurrence with an average time to recurrence of 45 months. There was no significant difference in recurrence rate between NTR and STR group. Mean growth free survival, though, was reached at 76 months for STR an not reached for NTR suggesting median growth free survival of over 100 months for NTR (Fig. 3).

**Fig. 4:**
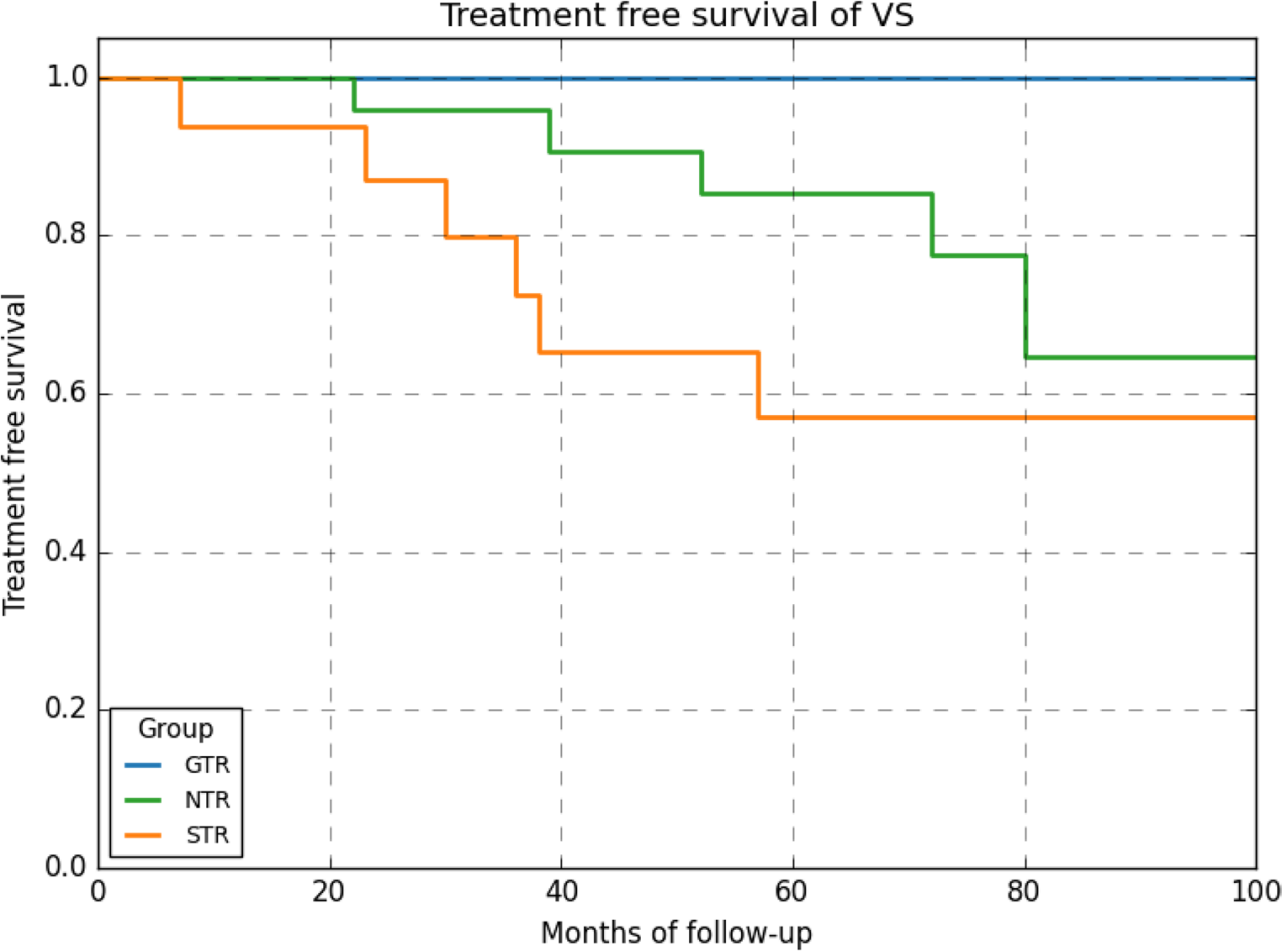
treatment of recurrence over time in months based on extent of resection; GTR versus NTR and STR. No statistical significant difference was found between STR and NTR

Of the 16 patients who developed a recurrence, 12 patients received additional treatment (10 SRS and 2 re-surgery). Patients with NTR had a longer treatment free survival then those with STR, but this was not statistically significant (Fig. 4).

## Discussion

In the treatment of patients with large CPA tumors, the principal goal is to achieve maximal resection while preserving FN function. The present study demonstrates that the use of IOM, specifically TcMEP may assist in preserving FN function and resulted in acceptably low recurrence rates.

### Facial nerve function

After one year, 83% of patients reported a good facial nerve outcome (HB grade I or II), only 5% of patients with VS had poor FN function (defined as HB grade V or VI). Moreover, a TcMEP threshold change of less than 20 mA always resulted in good FN outcome (HB grade I or II) at one year follow up when preoperative FN function was good. Although series of large VS are scarce, our results are in line with other reports. Grinblat et al. (2020) described a relatively large series of 389 patients with 37% good FN outcome and poor in 12% [10]. Additionally, they reviewed fourteen studies in which good FN outcome ranged from 16 to 92% of cases and poor FN outcome ranged between 2 and 26% [11–16].

High incidences of poor facial nerve function are typically found in series that report high rates of GTR [11–16]), while high incidences of good facial nerve function are reported in those aiming more for partial resections [11–16].

### Residual tumor and recurrence

Definitions of resection rates vary among studies which makes comparison difficult [2, 10]. In our VS patients we achieved GTR (no remnant reported by surgeon and no remnant on post-op MR) in 15% of cases. In 54% the remnant was < 1 cc (defined as NTR) and 31% showed STR. Median tumor remnant was 0.4 cc. Mean follow-up was almost 7 years. In patients that underwent GTR (*n* = 8) no recurrence occurred during follow up. In patients with any residual tumor (NTR or STR) 34% developed a recurrence of whom 12 (26% of all patients with a remnant) received additional treatment. Median time until growth was 76 months for those with STR (remnants > 1 cc) and not reached for those with NTR. Median time to treatment was not reached. Even though out definition of STR and NTR differs, these findings are in line with the recent report of Stastna et al., who suggested that a near total resection with a remnant of 0.25 cc would be optimal [9]. 

Kocharyan et al. (2020) showed that EOR less than GTR was associated with 6.3 times higher risk of recurrence compared to GTR [17]. They showed long term PFS (5 to 10 years) of about 90% in case of GTR and 75% for STR [17]. This seems comparable to our results, even though the tumors we report on are much larger (median of 28 mm versus 36 mm).

Interestingly, all patients with GTR, and the majority (66%) of patients with any remnant (either STR or NTR) showed no growth at all over time. In accordance with the study of Jeltema et al. (2015), we advocate that the majority of patients with small remnant tumors do not need upfront additional treatment with radiotherapy [18]. Moreover, literature shows no increased relative risk of need for salvage therapy in STR compared to NTR [9, 17]. All in all, this would justify a policy of watchful waiting (except for NF II patients) after surgery for VS, only referring patients with growth of the tumor remnant for adjuvant SRS.

The limitations of the current study include a retrospective design with variable follow up between patients. Although the strength of this study is the relatively large cohort of patients with Koos grade 4 CPA tumors, patient population is too small to perform multivariate analysis. Also there is a lack of consensus regarding universally accepted definitions of NTR, STR, and recurrence/regrowth, which makes comparison with the available literature challenging. Furthermore some bias is introduced because we are guided by TcMEP during surgery, which results in a shift towards lower threshold changes.

Overall, in light of the postoperative HB score, the small tumor remnants, good growth free survival and low rate of recurrence, we advocate the use of TcMEP guided surgery. This finding has changed our clinical practice, as we now guide resections by TcMEP threshold change, and resection is only continued when threshold change is below 20 mA. Even though this results in relatively fewer gross total resections, our data supports the notion that patients with remnant tumors do not need upfront additional therapy. We would advocate adjuvant therapy only when follow up shows growth of tumor remnant and for all vestibular schwannoma remnants in NF II patients.

In case of remnant VS (in non-NF patients), we would recommend a watchful waiting policy with the first follow-up MRI between 3 and 6 months to detect unexpected rapid growth in time. Unfortunately, our cohort is too small to predict the extent of growth based on residual size or the EOR.

## Conclusion

Surgeons facing patients with large VS must balance decisions between facial nerve preservation and extent of resection. Since the introduction of stereotactic radiosurgery, the need for (gross) total resection is less urgent. Nonetheless smaller remnants lead to less recurrence, as we and other have shown. TcMEP guidance in surgery of CPA tumors can help achieve these small remnants while preserving good facial nerve outcome. Growth free survival of VS remnants in non NF II patients is over 8 years in our series, which suggests that post-op radiotherapy should not be advised in these patients until growth is shown at follow-up.

Overall, this study brings us closer to an optimal surgical strategy in Koos grade 4 VS with good facial nerve outcome and low recurrence.

## Data Availability

No datasets were generated or analysed during the current study.

## Abbreviations

BAEP: Brainstem auditory evoked potential
CPA: Cerebellopontine angle
CSF: Cerebrospinal fluid
DeltaMEP/ ΔMEP: TcMEP threshold change
DNS: Extent of resection
EOR: Extent of resection
FN: Facial Nerve
HB: House-Brackmann grade
GTR: Gross total resection
IOM: Intra-operative neuro monitoring
NF: Neurofibromatosis II related schwannomatosis
NTR: Near total resection
PFS: Progression free survival
RUMC: Radboud University Medical Center
SRS: Single-fractionated stereotactic radiosurgery
STR: Subtotal resection
TcMEP: Transcranial motor evoked potentials
VS: Vestibular schwannoma

## References

[1] Tos MCS, Thomsen J (1998) Clinical experience with vestibular schwannomas: epidemiology, symptomatology, diagnosis, and surgical results. Eur Arch Otorhinolaryngol 255(1):1–6 9592666 10.1007/s004050050012

[2] Carlson ML, Link MJ (2021) Vestibular schwannomas. N Engl J Med 384(14):1335–1348 33826821 10.1056/NEJMra2020394

[3] Pruijn IMJ, Waterval JJ, Ter Laan M, Temel Y, Pegge SAH, Postma AA et al (2023) Subclassification of the Koos grade 2 vestibular Schwannoma into 2a and 2b for individualized patient care: A validity and reliability study. Eur J Radiol 162:110799 37001257 10.1016/j.ejrad.2023.110799

[4] Zachem TJ, Bello A, Lee B, Luo E, Woo J, Yoo S et al (2025) Current state of intraoperative neuromonitoring of the facial nerve during skull base surgery: A systematic review. J Clin Neurosci 140:111525 40753669 10.1016/j.jocn.2025.111525

[5] Sarnthein J, Szelenyi A (2024) Standardizing intraoperative facial nerve motor evoked potentials. Clin Neurophysiol 167:209–210 39341016 10.1016/j.clinph.2024.09.013

[6] Hendriks T, Kunst HPM, Huppelschoten M, Doorduin J, Ter Laan M (2020) TcMEP threshold change is superior to A-train detection when predicting facial nerve outcome in CPA tumour surgery. Acta Neurochir (Wien) 162(5):1197–1203 32146526 10.1007/s00701-020-04275-z PMC7156349

[7] Sarnthein J, Hejrati N, Neidert MC, Huber AM, Krayenbuhl N (2013) Facial nerve motor evoked potentials during skull base surgery to monitor facial nerve function using the threshold-level method. Neurosurg Focus 34(3):E7 23451854 10.3171/2012.12.FOCUS12386

[8] Nakatomi H, Jacob JT, Carlson ML, Tanaka S, Tanaka M, Saito N et al (2017) Long-term risk of recurrence and regrowth after gross-total and subtotal resection of sporadic vestibular Schwannoma. J Neurosurg 133(4):1052–1058 28524795 10.3171/2016.11.JNS16498

[9] Stastna D, Macfarlane R, Mannion R, Axon P, Bance M, Donnelly N et al (2025) Near-total resection in sporadic vestibular schwannoma: is there a volumetric threshold for a win-win scenario? J Neurosurg 1–8 10.3171/2025.6.JNS242466 41072057

[10] Grinblat G, Dandinarasaiah M, Braverman I, Taibah A, Lisma DG, Sanna M (2021) Large and giant vestibular schwannomas: overall outcomes and the factors influencing facial nerve function. Neurosurg Rev 44(4):2119–2131 32860105 10.1007/s10143-020-01380-6

[11] Samii M, Gerganov VM, Samii A (2010) Functional outcome after complete surgical removal of giant vestibular schwannomas. J Neurosurg 112(4):860–867 19663543 10.3171/2009.7.JNS0989

[12] Mehrotra N, Behari S, Pal L, Banerji D, Sahu RN, Jain VK (2008) Giant vestibular schwannomas: focusing on the differences between the solid and the cystic variants. Br J Neurosurg 22(4):550–556 18803080 10.1080/02688690802159031

[13] Charpiot A, Tringali S, Zaouche S, Ferber-Viart C, Dubreuil C (2010) Perioperative complications after translabyrinthine removal of large or giant vestibular schwannoma: outcomes for 123 patients. Acta Otolaryngol 130(11):1249–1255 20443757 10.3109/00016481003762316

[14] Silva J, Cerejo A, Duarte F, Silveira F, Vaz R (2012) Surgical removal of giant acoustic neuromas. World Neurosurg 77(5–6):731–735 22120302 10.1016/j.wneu.2011.08.019

[15] Anaizi AN, Gantwerker EA, Pensak ML, Theodosopoulos PV (2014) Facial nerve preservation surgery for Koos grade 3 and 4 vestibular schwannomas. Neurosurgery 75(6):671–675 discussion 6–7; quiz 7 25181431 10.1227/NEU.0000000000000547

[16] Monfared A, Corrales CE, Theodosopoulos PV, Blevins NH, Oghalai JS, Selesnick SH et al (2016) Facial nerve outcome and tumor control rate as a function of degree of resection in treatment of large acoustic neuromas: preliminary report of the acoustic neuroma subtotal resection study (ANSRS). Neurosurgery 79(2):194–203 26645964 10.1227/NEU.0000000000001162

[17] Kocharyan A, Daher GS, Curry SD, Klimara MJ, Farrokhian N, Coleman S et al (2024) Outcomes of Near-Total and subtotal resection of sporadic vestibular schwannoma: A systematic review and Meta-Analysis. Otolaryngol Head Neck Surg 171(3):642–657 38822753 10.1002/ohn.823

[18] Jeltema HR, Bakker NA, Bijl HP, Wagemakers M, Metzemaekers JD, van Dijk JM (2015) Near total extirpation of vestibular Schwannoma with salvage radiosurgery. Laryngoscope 125(7):1703–1707 25583352 10.1002/lary.25115

